# What is the most effective non-pharmacological treatment for poor sleep quality in Chronic obstructive pulmonary disease patients? a systematic review

**DOI:** 10.1007/s11325-026-03692-1

**Published:** 2026-05-02

**Authors:** Gary Yeung, Hady Atef

**Affiliations:** School of Allied Health Professions and Pharmacy (SAHPaP), Keele University, Staffordshire, ST5 5BG UK

**Keywords:** Chronic obstructive pulmonary disease, Sleep quality, Non-pharmacological

## Abstract

**Background:**

Chronic obstructive pulmonary disease (COPD) features poor sleep quality due to nocturnal dyspnea and psychological distress, leading to increased hospital admissions and reduced quality of life. Considering patient preference and existing evidence, non-pharmacological intervention, i.e. cognitive behavioral therapy for insomnia (CBT-I), on sleep for COPD patients had its potential efficacy. However, current guidelines do not include sleep management as standard care, and there is a lack of comprehensive reviews on this topic. This systematic review hence evaluated the effectiveness of non-pharmacological treatments for poor sleep in COPD patients.

**Methods:**

Literature identification was conducted through MEDLINE, CINHAL PLUS, AMED and ScienceDirect under PRISMA guidance. Randomized controlled trials, cohort studies and cross-sectional studies regarding poor sleep quality among COPD population were retrieved. Abstract, title and full text screening were performed by the author and assessed by a second reviewer. Risk of bias and quality appraisal was done with JBI scale.

**Results:**

12 articles (783 patients) were identified, including 9 randomized controlled trials, 1 cohort study and 2 cross-sectional studies. The risk of bias (quality) was ranked: low (high) for 3, moderate (moderate) for 7 and high (low) for 2. Several interventions were identified (number of study): CBT-I (2) and cognitive behavioral therapy (1), pulmonary rehabilitation (PR) (2), progressive muscle relaxation technique (PMRT) (2), non-invasive ventilation (NIV) (3), relaxation exercise (RE) (1) and therapeutic touch (TT) (1). Significant improvement in sleep quality was reported in PMRT, TT and RE.

**Conclusion:**

PMRT, TT and RE showed improvement in sleep quality in COPD patients yet unable to rank their effectiveness as heterogeneous outcome measures across studies. No substantial evidence was capable to demonstrate effectiveness of CBT-I, CBT, PR and NIV to explicit COPD patients. Moreover, future research including combination of non-pharmacological treatments is needed.

## What is new?

Cognitive Behavioural Therapy for Insomnia (CBT-I), Cognitive Behavioural Therapy (CBT), Pulmonary Rehabilitation (PR), and Non-invasive Ventilation (NIV) lack robust evidence for sleep improvement in COPD, despite their use in other populations.

Multimodal (combination) therapies may offer greater promise for this population.

Further high-quality research is needed to inform clinical guidelines.

## Introduction

Chronic Obstructive Pulmonary Disease (COPD) is a severe chronic respiratory disease. More than 3 million deaths worldwide have been attributed to COPD [1, 2]. COPD affects an estimated 11.7% of the global population [3]. COPD is a lung condition consisting of chronic bronchitis and emphysema [4]. The abnormal inflammatory response caused by these conditions impairs lung mechanics and gas exchange, leading to dyspnea due to inadequate ventilation, hyperinflation, forced vital capacity (FVC) and forced expiratory volume in the first second (FEV_1_) reduction and *V*′/*Q*′ mismatch [5–8]. These pathophysiological changes can induce agitated distress and fear in COPD patients, resulting in anxiety [9]. In addition, it is believed that periventricular lesions induced by hypoxia and systemic inflammation associated with COPD may be contributing factors of depression [10].

Approximately 85% of COPD patients are likely to develop anxiety [11]. Anxiety and depression are very common among COPD patients, with prevalence rates of 48.9% and 34.7%, respectively [12]. Additionally, Shah et al. [13] highlighted a close relationship between anxiety, depression and sleep, noting that unfavorable Hospital Anxiety and Depression Scale (HADS) score were associated with poor sleep quality. About 70% of COPD patients who have clinically diagnosed anxiety and depression report poor sleep quality [14]. Poor sleep is often linked to fear of death and the suffocating sensation during acute dyspnea exacerbations, as well as pessimistic thoughts, which further contribute to decreased sleep quality [15, 16]. In addition to psychological distress, sleep quality deterioration is also linked to nocturnal hypoxemia, resulting from exacerbated dyspnea symptoms while sleeping in supine position [17]. McNicholas et al. [18] noted that a decrease in respiratory drive and respiratory muscle hypotonia during non-REM (NREM) and REM sleep leads to hypoventilation. This reduces functional residual capacity (FRC), increases airway resistance, and contributes to further V′/Q′ mismatch, in addition to the existing hyperinflation seen in COPD, ultimately resulting in hypoxemia [18].

A common characteristic observed in COPD patients is diminished sleep efficiency and poor scores on the Pittsburgh Sleep Quality Index (PSQI) [19, 20]. Research defines poor sleep quality as sleep efficiency below 85% and a PSQI score above 5 [21, 22]. Notably, 38% of COPD patients reported PSQI scores above 5, with an average sleep efficiency of 66% [23, 24]. Importantly, the relationship between COPD and sleep quality is bidirectional [21–24]. COPD pathophysiology — including nocturnal hypoxemia, reduced respiratory drive, and respiratory muscle hypotonia — contributes to sleep disturbances (17,18). In turn, poor sleep quality has been associated with increased risk of acute exacerbations, hospital admissions, and mortality [25–28]. For example, Geiger-Brown et al. [25] and Shorofsky et al. [26], found that patients with high PSQI scores and sleep efficiency below 85% were more likely to experience exacerbations. Additionally, poor sleep quality negatively affects health-related quality of life (HrQOL) in COPD patients [29–31], and is commonly associated with insomnia (29.5%), obstructive sleep apnea (29.1%), and restless leg syndrome (21.6%) [32]. These findings highlight the need to address sleep disturbances as part of comprehensive COPD care.

In terms of management, patients often prefer non-pharmacological interventions due to concerns or negative perceptions about medications [33]. A current study by Kapella et al. (34) suggested that cognitive behavioral therapy for insomnia (CBT-I) is a feasible approach to COPD patients with insomnia given that CBT-I can improve emotional arousal and negative sleep belief. It was also suggested as an effective intervention for insomnia in COPD patients [34–36].

Moreover, in recent available researches, several holistic approaches, such as progressive muscle relaxation techniques (PMRT), non-invasive ventilation (NIV), pulmonary rehabilitation (PR), and therapeutic touch, have been reported to have beneficial effects on poor sleep quality in COPD patients [37–48].

Interestingly, given the above evidence suggesting the potential efficacy of non-pharmacological intervention for sleep disorders in COPD patients, the Global Initiative for Chronic Obstructive Lung Disease (GOLD) has not included routine sleep management apart from Obstructive sleep apnoea (OSA), despite the diversity of the sleep disorders within the COPD patients [2, 49]. Furthermore, there is currently no comprehensive review that summarizes and analyzes all existing evidence on non-pharmacological interventions specifically for this population. There is a need to review all available evidence to identify the most effective non-pharmacological interventions for poor sleep quality in COPD patients. This review may help guide future evidence-based practices for managing poor sleep quality in COPD.

Therefore, this study aims to systematically evaluate non-pharmacological interventions for poor sleep in the COPD patients to identify the most effective approach for improving sleep quality in this population. This will be achieved by conducting a systematic review of currently available studies, with a focus on a holistic approach.

## Method

The study design of this article is a systematic review performed under the guidance of the PRISMA (Preferred Reporting Items of Systematic Review and Meta-analysis) reporting guidelines 2020 [50].

Before implementing the literature identification methodology, the author registered this review protocol with PROSPERO under registration number CRD42024543251. A Student Project Ethics Committee (SPEC) notification was sent as required by library-based studies requirement.

A systematic identification study was conducted using four databases: MEDLINE, CINAHL PLUS, AMED, and ScienceDirect, as other databases yielded no results during the preliminary search. Moreover, the PICO search strategy with medical subject headings and keywords (Appendix 1) was implemented in all databases to ensure the search could identify research related to non-pharmacological interventions for COPD patients with poor sleep quality. Since a maximum of 8 Boolean terms being allowed in ScienceDirect, PICO was simplified for the search (Appendix 1). Filters were applied to ScienceDirect search only from 2008 to 2024, research articles, open access, and English only. The recently updated database search was performed on October 1, 2024.

### Inclusion and exclusion criteria

The inclusion criteria of this review: (1) COPD patients with poor sleep quality or insomnia or OSA or Restless Leg syndrome (RLS) (to include multiple sleep disorders with poor sleep quality); (2) non-pharmacological sleep interventions (i.e. CBT-I, PR, PMRT, breathing exercise, stimulus control therapy, sleep restriction therapy, RE, sleep hygiene, auricular acupressure, auricular acupuncture and therapeutic touch); (3) sleep quality outcome, including PSQI, Insomnia Severity Index (ISI), sleep efficiency, sleep latency, subjective sleep quality, COPD and Asthma Sleep Impact Scale (CASIS) and Richards-Campbell Sleep Questionnaire (RCSQ); (4) sleep quality as primary or secondary objectives; (5) peer-reviewed; (6) Randomized control trial (RCT), cohort study, case-control, case series, case report and cross-sectional study as included study type due to insufficient articles identification to generate a robust review if solely included RCTs.

The exclusion criteria were studies that were (1) either COPD patients without poor sleep quality or insomnia or OSA or RLS or poor sleep quality or insomnia or OSA or RLS patients without COPD; (2) pharmacological sleep interventions; (3) published before 2008; (4) translated into English or non-English text; (5) unable to gain full access; (6) systematic review, meta-analysis and quasi-experiment study types.

### Study selection

Mendeley Reference Manager [51] was selected as the primary reference software, importing all literature identified from databases. Duplicated articles revealed by Mendeley Reference Manager were removed by de-duplications [52], followed by a title, abstract and full-text screening to exclude irrelevant articles by inclusion and exclusion criteria for eligibility. All inclusion or exclusion reasons of studies at full-text screening were recorded in the study selection tool on Excel. Additionally, a second reviewer was recruited to independently conduct title, abstract, and full-text screening to ensure the eligibility of included articles. A third reviewer was available to make a final decision in case of controversy. Apart from Excel, the PRISMA flow diagram was recruited to record the whole study selection to track the number of articles in each screening stage and the reason for exclusion [50].

### Quality appraisal

The quality appraisal and risk of bias (RoB) assessment of selected literatures were assessed by the Joanna Briggs Institute (JBI) scale for RCT, cohort study and cross-sectional study [53], which consisted of 13 questions for RCT, ten questions for cohort study and eight questions for cross-sectional study. According to Barker et al. [54], the author could influence how the results of the Risk of Bias (RoB) for the articles are interpreted. If the score was higher than 70%, it would be considered as high quality and low RoB, 50%−69% as moderate quality and RoB, and low quality and high RoB if articles scored below 49%, based on previous systematic reviews [55–57]. The RoB process was reviewed by the second reviewer, with the third reviewer being involved only in case of differing opinions.

### Data extraction

The included studies were imported into two data extraction tools that categorized the studies based on their non-pharmacological methods. The first tool provided an overview of the included articles, extracting information such as author(s), year of publication, study type, interventions, treatment and comparison group, and the sleep quality outcome measures used as data. The second tool extracted continuous variables of sleep quality outcome measures, including PSQI, ISI, sleep efficiency, sleep latency, subjective sleep quality, CASIS, and RCSQ [58, 59].

### Statistical analysis

Given the heterogeneity in interventions, treatment techniques, control groups, and outcome measures in the included articles, it was not feasible to calculate weighted pooled estimates for each non-pharmacological intervention, indicating meta-analysis was not an option for this review [60]. All the outcome differences were presented as median [interquartile range] or mean ± standard deviation. The P values for each study outcome were presented with footnotes if the author reported any within-group or between-group differences for this review.

## Results

### Study identification

**Fig. 1:**
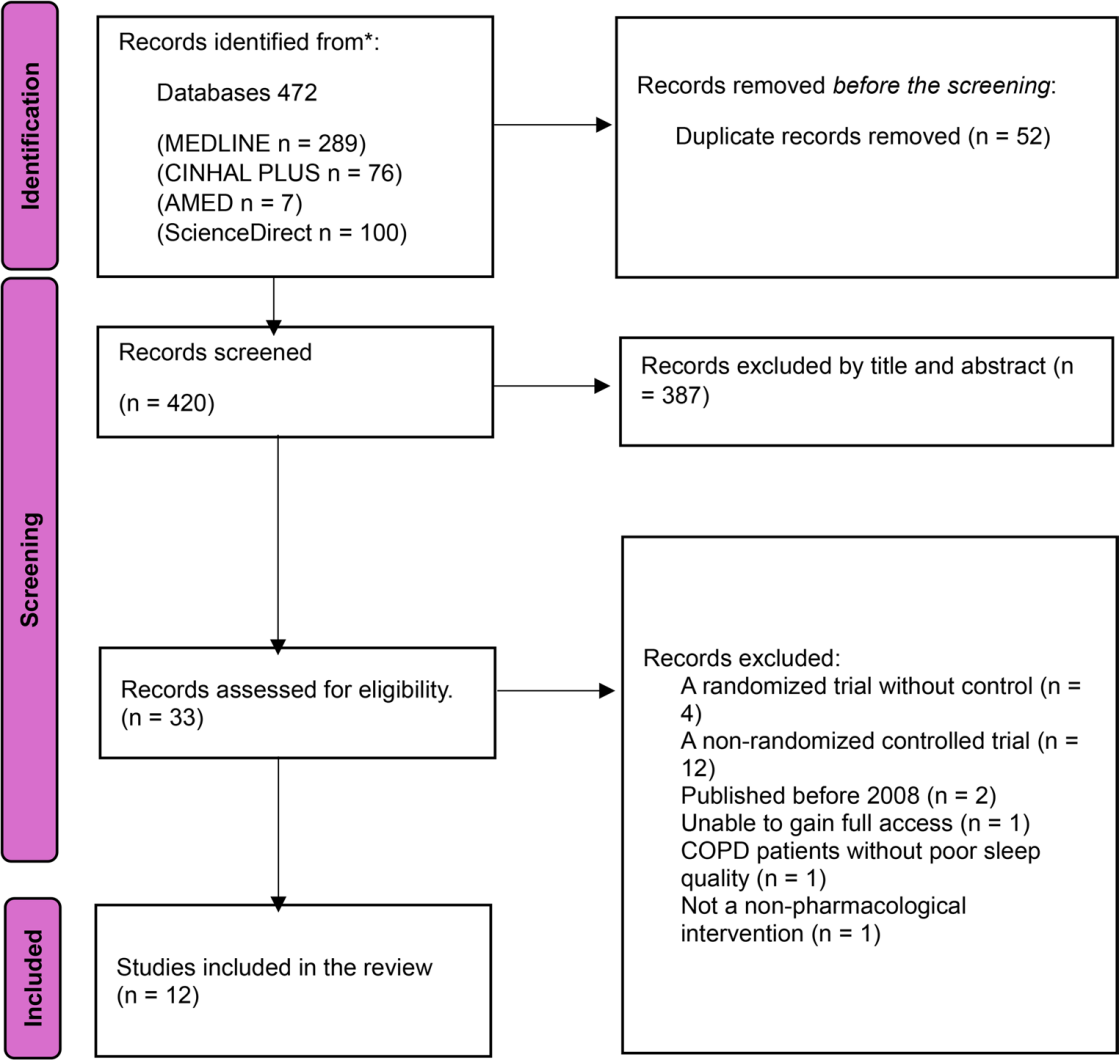
PRISMA (Preferred Reporting Items of Systematic Review and Meta-analysis) flow chart for literature identification procedure

472 studies were found in the databases in total. After eliminating 52 duplicate studies, 420 were left for the initial screening by their titles and abstracts. Of these, 387 studies were excluded, leaving 33 articles for further eligibility screening (Fig. 1). Out of the 33, 16 were excluded because of their study types (Table 1). Two articles were excluded because they did not focus on sleep quality in the COPD population or did not relate to the non-pharmacological intervention. Additionally, 2 articles were considered outdated, and 1 could not be accessed fully. Detailed reasons for exclusion can be found in Table 1. 12 articles were included for data analysis in this review after eligibility screening (Fig. 1).

**Table 1:** Excluded articles list (author and year) and exclusion reason

Article [Author(s), Year of publication]	Exclusion reason
Kapella et al., 2022	A randomised trial without control
Nilius et al., 2017	A randomised trial without control
Crisafulli et al., 2009	A randomised trial without control
Majorski et al.,2021	A randomised trial without control
Lan et al., 2014	A non-randomised controlled trial
Nobeschi et al., 2020	A non-randomised controlled trial
Akgün Şahin and Dayapoğlu, 2015	A non-randomised controlled trial
Gabrovska et al., 2023	A non-randomised controlled trial
Oh, Kim and Kim, 2016	A non-randomised controlled trial
McDonnell et al., 2014	A non-randomised controlled trial
Thapamagar et al., 2021	A non-randomised controlled trial
Yüksel et al., 2020	A non-randomised controlled trial
Xia et al., 2023	A non-randomised controlled trial
Franke et al., 2021	A non-randomised controlled trial
Sun, Shen and Shen, 2021	A non-randomised controlled trial
Ünal Aslan and Çetinkaya, 2024	A non-randomised controlled trial
Krachman et al., 1997	Published before 2008
Elliot et al., 1992	Published before 2008
Chabowski et al., 2019	Unable to gain full access
Wollsching-Strobel et al., 2022	COPD patient without poor sleep quality
Neale et al., 2022	Not a non-pharmacological intervention

### Included study overview

In total there were 12 eligible articles included in the analysis, consisting of 2 CBT-I [35, 61], 1 CBT [62], 2 PR [40, 63], with Deering et al. [63] included additional acupuncture, 2 PMRT [43, 45], 3 NIV (64–66), 1 RE [67] and 1 TT [46]. A total of 788 COPD patients aged 18 to 80 were involved in non-pharmacological interventions to treat poor sleep quality. Only 4 studies [35, 43, 45, 61] specified poor sleep quality in their inclusion criteria for participants. However, the COPD population in the remaining 8 studies [40, 46, 62–64, 66–68] were classified as having poor sleep quality based on their baseline measures which met the criteria of poor sleep quality: PSQI > 5, low sleep efficiency ≤ 85% or RCSQ < 70 [21, 22, 69]. All included studies assessed the effects on sleep quality as the primary or secondary objective (see Table 2).

**Table 2:** Overview of the included articles with regards to non-pharmacological intervention

Article [Author(s), Year of publication]	Study type	Non-pharmacological intervention	Intervention group		Control group	Study population	Sleep quality outcome measure
Kapella et al., 2011	Randomised controlled trial	Cognitive Behavioural Therapy for insomnia (CBT-i)	6-week sessions CBT-i		6-week sessions wellness education	Mild to severe and stable COPD patient (age ≥45) with poor sleep or insomnia	PSQI, Sleep efficiency (%), Sleep latency (min), ISI
Jun et al., 2024	Cross-sectional study	Cognitive Behavioural Therapy for insomnia (CBT-i)	6-week sessions CBT-I, CBTI + COPD-ED, COPD-ED or AC Low burden (Class 1) Intermediate burden (Class 2) High burden (Class 3)			Mild to severe and stable COPD patients (age ≥45) with poor sleep or insomnia (ISI ≥ 8)	ISI
Hynninen et al., 2010	Randomised controlled trial	Cognitive Behavioural Therapy for Anxiety and Depression (CBT)	7-week sessions CBT	7-week sessions enhanced standard care		COPD patients (age ≥40) with Beck Anxiety Inventory score (BAI) ≥15 and Beck Depression Inventory score (BDI) ≥ 13 PSQI >5②	PSQI, sleep efficiency (%)
Deering et al., 2011	Randomised controlled trial	Pulmonary rehabilitation (PR) + Acupuncture (A)	**(1)** 7-week sessions PR + 7-week sessions acupuncture **(2)** 7-week sessions PR		No intervention	Stable COPD patients without previous PR Sleep efficiency < 85%②	Sleep efficiency (%)
Cox et al., 2019	Cross-sectional study	Pulmonary rehabilitation (PR)	8-week sessions PR (centre and home based) completers			Stable COPD patients without previous PR for last 2 years Sleep efficiency < 85%②	Sleep efficiency (%), Sleep latency (min)
Işıkel H et al., 2023	Randomised controlled trial	Relaxation exercise	6-week sessions relaxation exercises		6-week sessions breathing exercises	COPD patients without previous PR PSQI >5②	PSQI, sleep efficiency ①, sleep latency ①, Subjective sleep quality ①
Article [Author(s), Year of publication]	Study type	Non-pharmacological intervention	Intervention group		Control group	Study population	Sleep quality outcome measure
Yilmaz and Kapucu, 2017	Randomised controlled trial	Progressive Muscle Relaxation Technique (PMRT)	8-week sessions PMRT		No intervention	Moderate to severe and stable COPD patients with self-reported insomnia	CASIS
Seyedi Chegeni et al., 2018	Randomised controlled trial	Progressive Muscle Relaxation Technique (PMRT)	8-week session PMRT		8-week sessions standard nursing care	Moderate to severe stable COPD patients with poor sleep (PSQI = 21) and Fatigue Severity Scale (FSS) ≥36	PSQI, sleep latency ①, Subjective sleep quality ①
Dreher et al., 2011	Randomised controlled crossover trial	Non-invasive ventilation (NIV)	2-night sessions crossover high-intensity non-invasive positive pressure ventilation (HI-NPPV)		2-night sessions crossover low-intensity non-invasive positive pressure ventilation (LI-NPPV)	Severe stable COPD patients with hypercapnic respiratory failure and on long term oxygen therapy Sleep efficiency < 85%②	Sleep efficiency (%)
McEvoy et al., 2009	Randomised controlled trial	Non-invasive ventilation (NIV)	6-monthly follow up NIV + usual care + long-term oxygen therapy		6-monthly follow up usual care + long-term oxygen therapy	Severe stable COPD patients (age<80) with hypercapnic respiratory failure and on long-term oxygen therapy Sleep efficiency < 85%②	Sleep efficiency (%)
Jolly et al., 2021	Cohort study	Non-invasive ventilation (NIV)	9.2 (6.0 to 13.5) weeks follow up after NIV initiation		N/A	COPD patients PSQI >5②	PSQI
Çalışkan and Cerit, 2021	Randomised controlled trial	Therapeutic touch (TT)	4-day sessions therapeutic touch + standard nursing care		4-day sessions standard nursing care	COPD patients(age>18) without use of sleep medications RCSQ <70②	RCSQ

*CASIS* COPD and Asthma Sleep Impact Scale, *COPD* Chronic Obstructive Pulmonary Disease, *PSQI* Pittsburgh Sleep Quality Index, *RCSQ* Richard-Campbell Sleep Questionnaire.

① PSQI subscale score

② Poor sleep criteria met for study inclusion

In this systematic review, there were 8 randomized control trials (66.7%), 1 randomized controlled crossover trial (8.3%), 1 cohort study (8.3%), and 2 cross-sectional studies (16.7%). Additionally, 3 studies were identified as having high quality and low RoB (25%), 4 as having moderate quality and RoB (58.3%), and 2 as having low quality and high RoB (16.7%; see Table 3). Data of all the included studies were listed in Table 4.

**Table 3:**
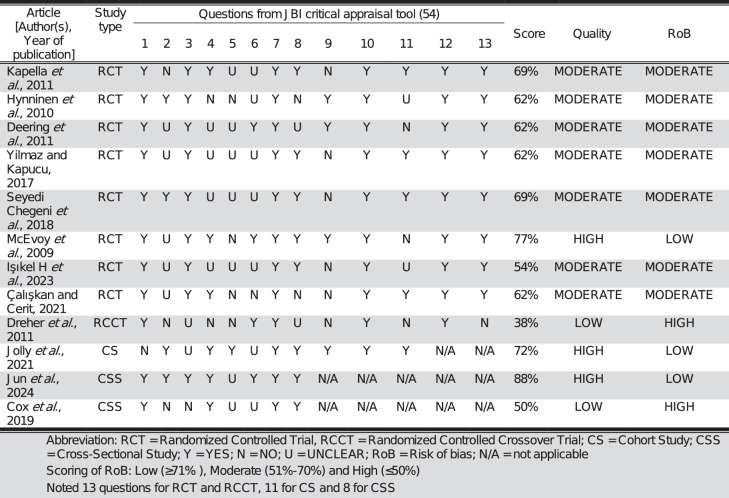
Risk of Bias and Quality Appraisal of included articles by JBI critical appraisal tools

*RCT* Randomized Controlled Trial, *RCCT* Randomized Controlled Crossoverr Trial, *CS* Cohort Study, *CSS* Cross Sectioonal Study, *Y* Yes, *N* No, *U* UNCLEAR, *RoB* Risk of bias, *NA* not applicable Scoring of RoB: Low (≥71%), Moderate (51% 70%) and High (≤50%) Noted 13 questions for RCT and RCCT, 11 for CS and 8 for CSSR

**Table 4:** Risk of Bias and Quality Appraisal of included articles by JBI critical appraisal tools

Article [Author(s), Year of publication]	Sample size, Intervention, Group			Sleep quality								
				PSQI			Sleep efficiency (%)					
				Pre-intervention	Post-intervention	Follow-up	Pre-intervention	Post-intervention	Follow-up			
Kapella *et al*., 2011	Phase 2 n = 9	CBT-I	Sleep diary	10.1 ± 3.7	7.2 ± 3.7	N/A	78 ± 15	88 ± 7①③	N/A			
			Actigraphy				71 ± 11	78 ± 11				
	Phase 2 n = 9	Wellness education	Sleep diary	7.1 ± 2.4	7.5 ± 2.4		82 ± 13	87 ± 5				
			Actigraphy				61 ± 17	64 ± 15				
Jun *et al*., 2024	n = 19	CBT-I, CBTI + COPD-ED, COPD-ED, AC	Class 1 (Low symptom burden)	N/A			N/A					
	n = 47		Class 2 (Intermittent symptom burden)	N/A			N/A					
	n = 25		Class 3 (High symptom burden)	N/A			N/A					
Hynninen *et al*., 2010	n = 25	CBT		9.8 ± 4.4	9.5 ± 3.5	8.8 ± 3.6	86 ± 9.5	86.4 ± 7.4	89.3 ± 6.7①③			
						(6-month)			(6-month)			
	n = 26	Enhanced standard care		8.4 ± 4.2	9 ± 4.5	8.6 ± 3.9	89.9 ± 7.8	89.9 ± 8.2	88 ± 7.2			
						(6-month)			(6-month)			
Cox *et al*., 2019	n = 48	PR		N/A			75 [66 to 84]	77 [67 to 87]	N/A			
Deering *et al*., 2011	n = 16	PR+A		N/A			76.2 ± 8.4	75.6 ± 10.8	76.1 ± 7.4			
									(3-month)			
	n = 25	PR					79.4 ± 10.7	81.4 ± 7.4	78.0 ± 9.3			
									(3-month)			
	n = 19	No intervention					74 ± 10.5	75.7 ± 10.6	N/A			
Yilmaz and Kapucu, 2017	n = 34	PMRT		N/A			N/A					
	n = 34	No intervention										
Seyedi Chegeni *et al*., 2018	n = 45	PMRT		6.33 ± 3.41	3.37 ± 2.36①⑤	N/A	N/A					
	n =46	Standard nursing care		6.41 ± 3.12	5.58 ± 2.94①⑤							
Dreher *et al*., 2011	n = 6	HI-NPPV		N/A	71.3 ± 19.3		87.4 ± 7.3	N/A	N/A			
	n = 7	LI-NPPV			80.8 ± 9.2		70.0 ± 21.5					
McEvoy *et al*., 2009	n = 41	NIV		N/A	59.6 [55.4 to 63.8]		59.7 [55.7 to 63.7]	N/A	N/A			
	n = 69	LTOT + usual care			61.0 [56.8 to 65.2]	N/A	N/A					
Jolly *et al*., 2021	n = 66	NIV		10 [5.5 to 14]	―1 [―6 to 0.8]⑥	N/A	N/A	N/A				
Işıkel H *et al*., 2023	n = 34	Relaxation exercise		9 [5 to 12]	6 [3 to 8]①⑤②③		1 [0–3]	1 [0–1]①③	N/A			
	n = 33	Breathing exercise		8 [5 to 12]	8 [5 to 11]①③		1 [0–2]	1 [0–1]				
Çalışkan and Cerit, 2021	n = 50	TT		N/A	N/A			N/A				
	n = 50	Standard nursing care										
Article [Author(s), Year of publication]	Sleep quality											
	Sleep latency (min)			ISI			Subjective sleep quality		CASIS		RCSQ	
	Pre-intervention	Post-intervention	Follow-up	Pre-intervention	Post-intervention	Follow-up	Pre-intervention	Post-intervention	Pre-intervention	Post-intervention	Pre-intervention	Post-intervention
Kapella *et al*., 2011	37 ± 60	19 ± 19	N/A	15.9 ± 5.6	10.7 ± 4.4①④	N/A	N/A		N/A		N/A	
	N/A	N/A										
	22 ± 18	23 ± 11		13.9 ± 4.5	12.4 ± 3.3							
	N/A	N/A										
Jun *et al*., 2024	N/A			13 ± 4⑤	7 ± 5⑤	8 ± 5⑤	N/A		N/A		N/A	
						(3month)						
	N/A			15 ± 3⑤	12 ± 4⑤	11 ± 5⑤	N/A		N/A		N/A	
						(3-month)						
	N/A			20 ± 4⑤	16 ± 5⑤	18 ± 6⑤	N/A		N/A		N/A	
						(3-month)						
Hynninen *et al*., 2010	N/A			N/A			N/A		N/A		N/A	
	N/A			N/A			N/A		N/A		N/A	
Cox *et al*., 2019	20 [8 to 39]	15 [6 to 28]	N/A	N/A			N/A		N/A		N/A	
Deering *et al*., 2011	N/A			N/A			N/A		N/A		N/A	
Yilmaz and Kapucu, 2017	N/A			N/A			N/A		57.14 ± 15.4	35.29 ± 13.8①③②③	N/A	
									60.08 ± 20.1	64.91 ± 16.1		
Seyedi Chegeni *et al*., 2018	1.60 ± 1.26	0.82 ± 0.93①⑤②⑤	N/A	N/A			1.20 ± 0.72	0.53 ± 0.54①⑤②⑤	N/A		N/A	
	1.80 ± 1.08	1.50 ± 0.98①⑤					1.30 ± 0.62	1.21 ± 0.55				
Dreher *et al*., 2011	N/A			N/A			N/A		N/A			
McEvoy *et al*., 2009	N/A			N/A			N/A		N/A			
Jolly *et al*., 2021	N/A			N/A			N/A		N/A			
Işıkel H *et al*., 2023	1 [0 to 2]	1 [0 to 2]①③	N/A	N/A	1 [1 to 2]	1 [1 to 1]①④②⑤	N/A		N/A			
	2 [1 to 3]	2 [1 to 2]			1 [1 to 2]	1 [1 to 2]						
Çalışkan and Cerit, 2021	N/A			N/A			N/A		36.52 ± 4.89①	44.98 ± 6.52①⑤②⑤	54.32 ± 7.47①⑤②⑤	66.18 ± 9.45①⑤②⑤
									(Day 1)	(Day 2)	(Day 3)	(Day 4)
									39.56 ± 6.39①	32.06 ± 7.89①	27.13 ± 8.61①	25.40 ± 10.34
									(Day 1)	(Day 2)	(Day 3)	(Day 4)

*CBT-I* congitive behavioral therapy for insomnia, *CBT* congitive behavioral therapy for anxiety and depression,* COPD-ED* COPD education, *AC* attention control, *PR* pulmonary rehabilitation, *A* acupuncture, *PMRT* progressive muscle relaxation technique, *HI-NPPV* high intensity noninvasive positive pressure ventilation, *LI-NPPV* low intensity noninvasive positive pressure ventilation, *NIV* non-invasive ventilation, *TT* therapeutic touch, *N/A* Not applicable, *CASIS* COPD and Asthma Sleep Impact Scale, *PSQI* Pittsburgh Sleep Quality Index, *RCSQ* Richard-Campbell Sleep Questionnaire

① Within-group significant difference; ② Between group significant difference; ③ p < 0.05; ④ p < 0.005; ⑤ p < 0.001; e.g. ②⑤ = between group difference, p < 0.001; ①④②⑤ = Within-group difference, p < 0.05 and between group difference, p < 0.001. If difference mentioned without p value in the articles, ③④⑤ would not be shown after ①②

⑥ Change in PSQI score post intervention. Noted no post-intervention PSQI was mentioned in original article

### Cognitive behavioral therapy for insomnia

In studies by Kapella et al. [35] and Jun et al. [61], participants diagnosed with mild to severe COPD and aged above 45 reported poor sleep quality/insomnia. In the Kapella et al. [35] study, the CBT-I group (*n* = 9) showed significant within-group improvement after six weeks of intervention sleep efficiency in sleep diary (from 78 ± 15 to 88 ± 7; *p* < 0.05) and ISI (from 15.9 ± 5.6 to 10.7 ± 4.4; *p* < 0.005) [61]. Although no significant improvement was shown in wellness education group (*n* = 9), no between-group difference was reported by Kapella et al. [35]. In Jun et al. [61] study, participants were randomly assigned with CBT-I, CBT-I with COPD education, COPD education or attention control for six weeks, followed by dividing them into three groups: Class 1 (low symptoms burden; *n* = 19), Class 2 (intermittent symptoms burden; *n* = 47) and Class 3 (high symptoms burden; *n* = 25). ISI scores were significant difference (*p* < 0.001) in all three symptoms burden groups at 3 different time points (i.e. pre, post and 3-month after intervention), however, no within-group difference was analyzed in this study [35]. This leads to concerns regarding the efficacy of CBT-I intervention to this specific population.

### Cognitive behavioral therapy for anxiety and depression

In Hynninen et al. [62] study, 51 COPD participants over the age of 45 with poor sleep quality [PSQI > 5 [21] were recruited for a comparison between CBT and enhanced standard care, which includes standard care for COPD plus monitoring psychological status with telephone contact every 2 weeks, for 7 weeks. A significant improvement in mean sleep efficiency within CBT group (*n* = 25) was reported (89.3% ± 6.7%; *p* < 0.05) from after intervention to 6-month follow up yet moderate effect size was reported in between group effects (Cohen’s d = 0.4) compared to enhanced care group (*n* = 26) [62]. Also, there were no significant within-group differences in PSQI post CBT (Cohen’s d = 0.3) and 6-month follow-up (Cohen’s d = 0.3).

### Pulmonary rehabilitation

Cox et al. [40] and Deering et al. [63] conducted studies regarding effect on sleep quality in stable COPD patients who had not undergone PR before and had less than 85% sleep efficiency, by Jung et al. [22], following PR intervention. Cox et al. [40] conducted an 8-week PR study (*n* = 48) while Deering et al. [63] conducted a 7-week PR study (*n* = 25) compared to no intervention (*n* = 19) with post-intervention and 3-month follow-up. Furthermore, Deering et al. [63] also examined the potential effect of acupuncture as an additional therapy to PR by a 7-week PR plus acupuncture study (*n* = 16). No significant between- and within-group difference in sleep efficiency was found in post intervention in all groups after interventions by both studies [40, 63] as well as 3-month follow-up by Deering et al. [63]. Similarly, sleep latency was found no significant difference after PR by Cox et al. [40].

### Progressive muscle relaxation technique

Two articles by Yilmaz et al. [43] and Seyedi Chegeni et al. [45] examined the effects of PMRT intervention on sleep among moderate to severe stable COPD patients with poor sleep quality (i.e. self-report or PSQI = 21). Both studies compared 8-week PMRT intervention to either standard nursing care or no intervention [43, 45]. Yilmaz et al. [43] found a significant between-group and within-group reduction in mean CASIS (from 57.14 ± 15.4 to 35.29 ± 13.8; *p* < 0.05) in the PMRT group (*n* = 34) compared to no intervention group (*n* = 34). Furthermore, Seyedi Chegeni et al. [45] reported significant within- and between-group improvements in mean subjective sleep quality (1.20 ± 0.72 to 0.53 ± 0.54; *p* < 0.001) as PSQI subscale score in PMRT group (*n* = 45) compared to the standard nursing care group (*n* = 46). Although both PMRT group and standard nursing care group showed significant improvements (*p* < 0.001) in mean PSQI scores and sleep latency, PMRT group demonstrated a notable treatment effect with a significant between-group difference in mean sleep latency (1.60 ± 1.26 to 0.82 ± 0.93; *p* < 0.001) (45).

### Non-invasive ventilation

The effect of NIV, including continuous positive airway pressure (CPAP) of NIV, on sleep quality among COPD patients who received long-term oxygen therapy and sleep efficiency of less than 85% by Jung et al. [22] was investigated by McEvoy et al. [64], Dreher et al. [65], and Jolly et al. [66]. Dreher et al. [65] found no significant between-group difference in mean sleep efficiency among the High Intensity Non Invasive Ventilation (HI-NIV) (*n* = 6) and Low Intensity Non Invasive Ventilation (LI-NIV) group (*n* = 7) in a 2-night crossover session. Similarly, McEvoy et al. [64] reported that a 6-month NIV treatment did not result in significant within-group difference in mean sleep efficiency the intervention group (*n* = 41) compared to Long-term Oxygen Therapy (LTOT) plus usual care (*n* = 69). No within-group analysis was conducted by Dreher et al. [65] while no between-group analysis was performed by McEvoy et al. [64]. Similarly, Jolly et al. [66] reported no significant improvement in the median PSQI score change regarding 9.2-week follow-up of COPD cohort (*n* = 66) compared to other lung condition cohorts after NIV initiation.

### Relaxation exercises

67 COPD patients with poor sleep quality [PSQI > 5 by Mollayeva et al. [21] who had not previously undergone pulmonary rehabilitation were recruited in Işıkel et al. [67] study. The study found that after six weeks of RE (*n* = 34), the median PSQI score decreased significantly within the group (from 9 [5 to 12] to 6 [3 to 8]; *p* < 0.001) and between the groups (*p* < 0.05) when it compared to breathing exercise group (*n* = 33) (67). Additionally, within the PSQI subscale score, similar results were observed, by Işıkel et al. [66] study, in subjective sleep quality (from 1 [1 to 2] to 1 [1 to 1]; within-group, *p* < 0.005; between group, *p* < 0.001), while only significant within-group differences were found in sleep efficiency score (*p* < 0.05) and sleep latency score (*p* < 0.05).

### Therapeutical touch

In a study conducted by Çalışkan et al. [46], 100 COPD patients with poor sleep quality (defined as RCSQ < 70) were randomly assigned to either a 4-day TT intervention group (*n* = 50) or a standard nursing care group (*n* = 50). TT was delivered alongside standard care and sleep quality was assessed daily using the RCSQ. The TT group demonstrated progressive and statistically significant improvements in mean RCSQ scores from Day 1 (36.52 ± 4.89) to Day 4 (66.18 ± 9.45), with both within-group and between-group differences reaching significance (*p* < 0.001). These findings suggest a short-term benefit of TT on perceived sleep quality. However, as TT was evaluated in only one study, its generalisability remains limited and further research is needed to confirm its efficacy.

## Discussion

In this systematic review, 12 studies were selected to investigate non-pharmacological interventions for COPD patients with poor sleep quality. The main findings of the studies were as follows: (1) PMRT, TT, and RE demonstrated their effectiveness in non-pharmacological treatment. However, the most effective treatment could not be conclusively determined. (2) CBT-I, CBT, PR, and NIV were unable to prove their treatment effectiveness on sleep quality in COPD patients due to insufficient data analysis. (3) Additionally, combining non-pharmacological treatments may offer a more effective approach to improving sleep quality in COPD patients.

PMRT, TT, and RE have been identified as effective non-pharmacological approaches against COPD patients suffering from poor sleep quality [43, 45, 46, 67]. For instance, Seyedi Chegeni et al. [45] found significant improvements in sleep latency and subjective sleep among COPD patients who underwent PMRT compared to those receiving standard nursing care. Similarly, Yilmaz et al. [43] reported that PMRT significantly enhanced CASIS scores within the PMRT group compared to the control group with no intervention, indicating improved sleep quality in COPD patients.Işıkel et al. [67] also demonstrated that RE effectively improved both subjective sleep quality subscale score means and overall mean PSQI scores compared to a control group. In fact, the benefits of PMRT and RE on sleep quality in COPD patients were attributed to shared mechanisms. PMRT was believed to reduce the level of fatigue, pain, anxiety and dyspnea in COPD patients while RE decreased the severity level of dyspnea in COPD patients, ultimately leading to improved sleep quality in COPD patients [43, 45, 67]. Besides, PMRT might improve psychological wellbeing by building energetic and cheerful emotions, as well as providing mental and physical relief, which could further improve the dyspnea perception and sleep quality in COPD patients [43]. Additionally, TT has been shown its effectiveness in average sleep quality scores post-intervention compared to standard nursing care, as measured by the RCSQ, among COPD participants [46]. According to Çalışkan et al. [46], TT helped reduce anxiety and improve sleep quality by creating a relaxing and calming experience for COPD patients, given that anxiety has been linked to poor sleep quality in this population [12, 13]. However, the heterogeneity in outcome measures (i.e. PSQI, RCSQ, CASIS and sleep latency) [43, 45, 46, 67] has made it challenging to compare the result and determine the most effective non-pharmacological intervention for poor sleep quality in COPD patients.

CBT-I had long been recognized as evidence-based practices for treating insomnia [70]. However, CBT-I has not demonstrated significant benefits in improving sleep quality for patients with COPD in this study. Although Kapella et al. [61] demonstrated improvements in sleep efficiency and ISI following CBT-I in COPD patients, the findings may be biased due to insufficient between-group analyses that could demonstrate the advantages of CBT-I over standard care, a limitation acknowledged by the authors. Similarly, Jun et al. [35] did not perform within-group analyses regarding ISI over different time periods across the three intervention groups, making it challenging to confirm the effectiveness of CBT-I in enhancing sleep quality for COPD patients. Furthermore, according to the National Institute for Health and Care Excellence (NICE) [70], while CBT-I has been recommended for general insomnia, there is no substantial evidence regarding its effect on sleep quality specifically in COPD patients. The psychophysiological foundations of CBT-I aim to enhance homeostatic sleep drive to improve attitudes and beliefs about sleep [71]. This approach is hypothesized to improve sleep quality in COPD patients [34]. However, due to the multifactorial features regarding poor sleep quality in this population, such as nocturnal hypoxia, reduced respiratory drive, and diminished respiratory muscle tone, future research is required to confirm the efficacy and the holistic nature of the CBT-I for COPD patients [17, 18]. Conversely, given the significant relationship between anxiety and depression and poor sleep in COPD patients [15, 16], Hynninen et al. [62] reported that CBT was not an outstanding choice compared to standard nursing care regarding sleep quality improvement among COPD patients. The findings showed no significant between-group effects in both sleep efficiency and PSQI after CBT intervention compared to nursing care [62]. This was thought to be related to the sleep complaints, which often reflected actual fatigue and abnormal breathing during sleep, reported by COPD patients, suggesting that CBT alone may be insufficient to enhance sleep quality in this cohort [62].

On the other hand, NIV has failed to demonstrate improvements in poor sleep quality among COPD patients. McEvoy et al. [64] did not analyze between-group differences, and Dreher et al. [65] lacked a within-group difference analysis between the HI-NIV and LI-NIV groups. Both studies reported no significant improvement in sleep efficiency [64, 65]. Moreover, Jolly et al. [66] showed no significant difference in PSQI after intervention, failing to demonstrate that NIV was effective against poor sleep quality in COPD patient. While these results might contradicted the hypothesized mechanisms of NIV for addressing respiratory disadvantages associated with sleep in COPD patients, such as upper airway limitation by positive end-expiratory pressure, respiratory muscles hypotonia and nocturnal hypoventilation by inspiratory positive pressure [48], LI-NIV was expected to have no effect on sleep quality, whereas HI-NIV might only prevent further deterioration of sleep quality in COPD patients [67]. Additionally, McEvoy et al. [64] mentioned that the level of NIV used in their study might not sufficiently increase central respiratory drive in COPD patients, resulting in no treatment benefit on sleep quality in this population.

Besides, despite substantial evidence suggesting beneficial effects on poor sleep quality in COPD patients [37–39, 42], PR was unable to exhibit positive effective on sleep quality in this population. Cox et al. [40] demonstrated that PR has no significant improvement in sleep efficiency and sleep latency in this cohort while Deering et al. [63] showed similar findings in sleep efficiency as well, illustrating no treatment effect of PR regarding poor sleep quality in COPD patients compared to the group without intervention. This could be explained by the reduced daily physical activity capacity of COPD patients compared to normal participants in PR, leading to incapability to exercise at adequate intensity and improve sleep quality in COPD patients following PR [40]. In addition, even if COPD patients achieved sufficient intensity to improve exercise capacity following PR, no positive result on poor sleep quality was expected among them as there was no correlation between poor sleep and exercise capacity in COPD population [72].

Furthermore, Deering et al. [63] investigated the benefits of combining interventions by delivering acupuncture, which alleviates dyspnea, as an adjunct to PR and their results showed no improvement. This finding alone does not allow for a definitive conclusion about the feasibility of using a combination of non-pharmacological interventions to enhance sleep quality in patients with COPD, particularly since it was the only piece of evidence available to assess the benefits of combined treatment in this population [63]. Conversely, Hynninen et al. [62] suggested that the sleep quality of patients with COPD could improve by combining CBT with PR. This combined approach emphasizes psychological education alongside exercise and breathing techniques to alleviate anxiety and depression in this population [62]. This presents a potential comprehensive strategy for addressing the sleep quality of COPD patients.

### Implications

As mentioned, the heterogeneous outcome measures across the identified studies limited the ability to draw a conclusion of which non-pharmacological intervention is the most effective against poor sleep quality in COPD patients in this review. Further research should consider standardizing the selection of outcome measures used in sleep quality to enable the comparison regarding effectiveness of different interventions. Additionally, the studies revealed inconsistent definitions regarding poor sleep quality within the COPD population. It is crucial for future research to establish standardized definitions of poor sleep quality to ensure that study samples have a consistent baseline regarding this issue. Furthermore, many studies assessing CBT-I and NIV lacked robust statistical analyses, making it difficult to ascertain the effectiveness of these treatments. This implies the necessity of further research for comprehensive statistical analyses, such as within-group and between-group analysis, to enhance the result validity. Finally, exploring the combination of various non-pharmacological interventions to address poor sleep quality in COPD patients may be a promising therapeutic approach, warranting further investigation.

### Limitations

The level of strength regarding the conclusion from this review was limited due to the heterogeneity in RoB and the quality of the included articles, with most of the identified literatures in moderate quality and RoB (see Table 3). The absence of instructions and criteria for presenting quality outcomes following quality appraisal also highlighted the limitations of the JBI appraisal tool as well as the strength of this review [73]. Moreover, a limited number of literatures were identified considering the specificity of this project title on sleep quality in COPD patients and non-pharmacological interventions, indicating a negative effect on the strength of evidence regarding this review as well. A further limitation of this review is that COPD severity was not stratified according to the GOLD classification system. This decision was made due to inconsistent reporting across the included studies and the limited availability of subgroup data. As a result, the generalisability of the findings across all stages of COPD may be constrained. Nonetheless, the review provides a comprehensive synthesis of the available evidence and highlights the need for future studies to report and analyse outcomes by disease severity.

## Conclusions

Non-pharmacological treatments such as PMRT, TT, and RE demonstrated their effectiveness against poor sleep quality in COPD patients. However, the heterogenous outcome measures used across different studies make it difficult to rank their effectiveness, leading to an inability to conclude which is the most effective non-pharmacological intervention for poor sleep in this population. Additionally, CBT-I, CBT, NIV, and PR have not consistently demonstrated effectiveness in improving sleep quality for COPD patients, despite substantial evidence supporting their potential benefits in different populations. Furthermore, further research is needed to investigate potential therapeutic approaches that combine various non-pharmacological interventions to treat poor sleep quality in COPD patients. Future studies should also standardize the result analysis, outcome measures and definitions of poor sleep quality to allow for meaningful comparisons across research.

## Data Availability

Our manuscript has no associated data.

## Abbreviations

CASIS: COPD and Asthma Sleep Impact Scale
CBT-I: Cognitive Behavioural Therapy for Insomnia
COPD: Chronic Obstructive Pulmonary Disease
CPAP: Continuous Positive Airway Pressure
FEV1: Forced Expiratory Volume in the first second
FRC: Functional Residual Capacity
FVC: Forced Vital Capacity
GOLD: Global Initiative for Chronic Obstructive Lung Disease
HADS: Hospital Anxiety and Depression Scale
HI-NIV: High Intensity Non-Invasive Ventilation
HrQL: Health-related quality of life
ISI: Insomnia Severity Index
JBI: Joanna Briggs Institute
LI-NPPV: Low Intensity Non-Invasive Ventilation
LTOT: Long-term Oxygen Therapy
NICE: National Institute for Health and Care Excellence
NIV: Non-Invasive Ventilation
NREM: Non-REM
OSA: Obstructive Sleep Apnoea
PMRT: Progressive Muscle Relaxation Technique
PR: Pulmonary Rehabilitation
PRISMA: Preferred Reporting Items of Systematic Review and Meta-analysis
PSQI: Pittsburgh Sleep Quality Index
RCSQ: Richards-Campbell Sleep Questionnaire
RCT: Randomised controlled trial
RE: Relaxation Exercise
RLS: Restless Leg Syndrome
RoB: Risk of bias
SPEC: Student Project Ethics Committee
TT: Therapeutic Touch
